# Anti-Proliferative Activities of Sinigrin on Carcinogen-Induced Hepatotoxicity in Rats

**DOI:** 10.1371/journal.pone.0110145

**Published:** 2014-10-20

**Authors:** Meng Jie, Wan Man Cheung, Vivian Yu, Yanling Zhou, Pak Ho Tong, John W. S. Ho

**Affiliations:** School of Life Sciences, The Chinese University of Hong Kong, Shatin, Hong Kong; Duke University Medical Center, United States of America

## Abstract

Liver cancer is one of the leading causes of cancer death worldwide. A very high incidence of new liver cancer cases is diagnosed every year, and metastasis has been found to correlate to poor prognoses in humans. Better treatments for liver cancer are thus clearly needed. Sinigrin is one of the major ingredients present in Brassica nigra, which has been used in combination with other herbs for treatment of various diseases. The anti-proliferative activities of sinigrin were studied in a model of carcinogen-induced hepatotoxicity in rats. Rats were orally administered with sinigrin on a daily basis for three months before sacrifice. Sinigrin was found to significantly inhibit the proliferation of liver tumor cells; the number of surface tumors in the rat liver was dramatically reduced. Sinigrin induced apoptosis of liver cancer cells through up-regulation of p53 and down-regulation of Bcl-2 family members and caspases. Our findings indicated that the liver functions were gradually restored after treatment with sinigrin and that the agent did not cause liver toxicity. Cell cycle analysis indicated that sinigrin caused cell cycle arrest in G0/G1 phase. The results suggest that sinigrin exerts important anti-proliferative activities in carcinogen-induced hepatocarcinogenesis in rats, and highlight the potential of sinigrin as an anti-cancer agent for liver cancer.

## Introduction

Sinigrin is a glucosinolate present in the seeds of Brassica nigra and other Brassicaceae family including broccoli and Brussels sprouts. Glucosinolates have been reported to exhibit different pharmacological properties in vitro [1]–[4]. Sinigrin has been reported to exhibit anti-tumor activity [1]. The metabolic activation of sinigrin leads to the formation of isothiocyanates which are believed to contribute to the anti-tumor activity [2]–[4]. The therapeutic benefits of brassica vegetables and the anti-cancer activity of sinigrin in cancer cell lines are well established [5]–[7]. The studies suggested that sinigrin could inhibit the cancer cell growth. An in vivo study reported the effects of the glucosinolates on carbohydrate and lipid metabolism in the rat model [1], [8]. Glucosinolates increased total cholesterol level, whereas the triacylglycerol levels in blood were found to be lowered [8]. The glucosinolates are believed to lower the health risk of particular degenerative diseases [9]. Glucosinolates are hydrolyzed to yield isothiocyanates which are excreted in the urine as an N-acetyl-cysteine conjugates. Sinigrin may also cause an increase in the activity of quinone reductase and glutathione-S-transferase in rats [10]. However, the precise details of the pharmacological activity of sinigrin in rats are not currently available.

## Materials and Methods

Trypsin-EDTA (1X), Dulbecco’s Modified Eagle Medium (DMEM), RPMI Medium 1640, and PSN Antibiotic mixture were purchased from Invitrogen (CA, USA). Fetal Bovine Serum (FBS) was purchased from Biosera (UK). Xylene cyanole and ethidium bromide (EB), Dimethylsulfoxide (DMSO) and other chemicals & reagents were purchased from Sigma Chemicals (St. Louis, USA). Sinigrin with 98% purity was purchased from Sigma-Aldrich (USA). RNase A was purchased from Amersham (USA).

ALT/SGPT (UV-Rate) & AST/SGOT (UV-Rate) Kits for detection of alanine and aspartate transferases were purchased from Stanbio Laboratory (TX, USA). Anti-GST-P polyclonal antibody (rabbit) was purchased from Medical & Biological Laboratories. Biotinylated goat anti-rabbit IgG and avidinbiotin-peroxidase complex (ABC Staining System) were purchased from Santa Cruz Biotechnology (CA, USA).

### Neutral Red Assay

HepG2, WRL-68 and Clone 9 cells, that were raised in the culture flasks with complete culture medium, DMEM, were trypsinized and washed. Tens of thousands of cells were seeded in 96-well plates. After 24 hours pre-incubation, cells were treated with different concentrations of sinigrin and incubated for 72 hours. After incubation, cells were harvested and washed twice with 1X PBS buffer. Fifty microliters of Neutral Red solution was added to each well. The whole plate was placed into the incubator at 37°C with 5% CO_2_. After 1 hour incubation, the plate was washed with 1X PBS buffer twice and dried in a 60°C oven for overnight. One hundred microliters of a 1% SDS solution was added to each well to lyse the cells and resolve the Neutral Red dye. The color was measured at an OD of 540 nm.

### Cell Cycle Analysis

HepG2 cells were trypsinized, washed and seeded into the 25 mm^2^ culture plates with complete DMEM medium. After 24 hours of pre-incubation, different concentrations (250 µM, 500 µM) of sinigrin were added into the culture plates. Complete DMEM medium was added to one plate with cells as the control. After different periods of incubation with singrin, cells were harvested. The cell suspension solution was centrifuged at 1,000 rpm for 3 minutes. The supernatant was removed and the cell pellet resuspended in 1 ml 1X PBS buffer for washing. The suspension was transferred into a microcentrifuge tube. The washing solution was centrifuged at 1,000 rpm for 3 minutes. The supernatant was discarded. The cell pellet was resuspended in 1 ml 70% ethanol and 0.1 ml 1X PBS buffer. The suspension was kept at 4°C overnight to fix the cells. After centrifugation at 1,000 rpm for 3 minutes, 1 ml 1X PBS was added for washing. Then 1 mL PI solution was added to the cells and allowed to incubate at 37°C for 30 minutes. DNA content was analyzed using FACScan Flow cytometery with sufficient Shealth Fluid. The results were analyzed by FCS express software produced by De Novo Software Co.

### Animal Treatment

Sprague Dawley (SD) rats were induced with carcinogens in the in vivo experiments according to the previously established method [11]. This study was carried out in strict accordance with the recommendations in the Guide for the Care and Use of Laboratory Animals of the Chinese University of Hong Kong. The protocol was approved by the Committee on the Ethics of Animal Experiments of the CUHK (Permit Ref. No. 003/008). All efforts were made to minimize any suffering encountered by our subject animals.

Male SD rats (100–120 g body weight) were obtained from Laboratory Animal Services Center of The Chinese University of Hong Kong. The rats were housed in the rodent animal room with a 12 hour light-dark cycle and constant temperature of 25°C. Food and water were given ad libitum. All the animals were observed and weighed daily. Fifty rats were randomly divided into 3 groups, negative control (normal) group (10 rats), a positive control (untreated) group (10 rats) and three sinigrin-treatment groups (10 rats each). Sinigrin at 10 mg/kg, 15 mg/kg and 25 mg/kg was administered orally to the treatment groups on a daily basis for a duration of 20 weeks.

During the experiment, the body weight of each rat was monitored daily. At the end of the experiment, all rats were killed by carbon dioxide asphyxiation and the blood was collected. The rat liver was perfused with ice old 1X PBS and quickly removed, washed and weighted. The rat livers were histologically analyzed. The liver slices were fixed with 10% formaldehyde solution for 24 hours and stored in 75% ethanol for histological analysis.

### Measurement of Serum ALT and AST activities

Rat blood was collected in a 13 ml BD PLUS-SST II serum vacutainer and allowed to stand for 20 minutes on ice for coagulation. Vacutainers were then centrifuged at 3,500 rpm for 15 minutes. The upper layer serum samples were collected and AST/ALT assays were performed within 2 days to minimize loss of enzymes and their activities.

The serum AST activity assay was measured by using a Stanbio AST (UV-Rate) kit. 15 mL ddH_2_O were added into one bottle of reagent and mixed thoroughly according to the kit protocol. 1 mL of the reagent was incubated at 37°C for at least 10 minutes for each reaction. 100 µL of sample serum were added to the reagent solution and incubated for exactly 1 minute. Absorbance was measured at 340 nm. After incubation for 1 minute, readings were taken and the reading at this point was counted as time zero. Readings were then taken at 30 second intervals for a period of 3 minutes. The change of absorbance per minute was calculated and the AST activities could be calculated by the formula (U/L = ΔA/min×1768).

The ALT assay was measured using a Stanbio ALT (UV-Rate) kit. The protocol of the experiment was the same as for the AST assay.

### H&E Staining

Slides with liver sections after fixation were placed on a slide holder, dewaxed and rehydrated with xylene (3 times, each time 5 minutes), 100% ethanol (3 times, each time 3 minutes), 95% ethanol (3 minutes), 80% ethanol (3 minutes) and distilled water (5 minutes) in this particular order. Slides were then immersed in hematoxylin for about 3 minutes and allowed to develop the coloration for several minutes in water, followed by brief destaining with acidic ethanol and water. Slides were then immersed into eosin for about 30 seconds to stain cytoplasms in red and followed by washing in tap water for several minutes. Slide-checking was necessary to assure the best staining effect. Slides were then dehydrated with 95% ethanol (3 minutes), 100% ethanol (3 times, each time 5 minutes), and xylene (3 times, each time 5 minutes) in order. Finally, the slides were mounted with cover-slip and allowed to dry. An Axiophot-2 Universal microscope (Zeiss) connected with computer were used to capture cell contents images using Spot 32 image capture software (Diagnostic Instruments).

### GST-p Immuno-staining

Slides with liver sections were placed on a slide holder, dewaxed and rehydrated with xylene and different concentrations of ethanol as mentioned previously. Slides were incubated with 1% H_2_O_2_ solution for 5 minutes at room temperature to block endogenous peroxidase activity followed by washing in tap water for 5 minutes. Hot citric acid buffer (pH 6.0) was freshly prepared. Slides were heated in hot citric acid buffer for 5 minutes to ensure antigen retrieval. Slides were washed with three changes of PBS for 5 minutes each. The slides were removed from PBS and placed on a moist chamber. The immuno-staining was performed with a Santa Cruz ABC staining kit. 200 µL of diluted normal serum solution were added to the sections on each slide and allowed to incubate for 1 h to block unspecific binding. After draining the normal serum, 200 µL diluted Anti-GST-pi antibody (rabbit polyclonal, 1∶10) was then applied and incubated overnight at 4°C. After washing with 1X PBS for three times, 5 minutes each, 200 µL diluted biotinylated secondary antibody (1∶200) was applied and incubated for 30 minutes at room temperature. After three changes of PBS for 5 minutes each, 200 µL avidin & biotinylated horseradish peroxidase macromolecular complex (ABC) was added and allowed to incubate for 30 minutes. Slides were washed with PBS and visualized by DAB solution for approximately 3 minutes. After washing with tap water for several minutes, slides were counterstained in hematoxylin for 3 minutes and washed with tap water. Slides were dehydrated as mentioned previously. Finally, the slides were mounted with cover-slip and allowed to dry. The image of the slides were checked and captured by an Axiophot-2 Universal microscope (Zeiss) connected with a computer for documentation. The image was recorded by Spot 32 image capture software (Diagnostic Instruments). The image with 2.5 fold magnification was used to calculate the GST-p positive area/whole area ratio by Analysis software Image J.

### P53 and MDM2 mRNA expression by RT–PCR analysis

Three micrograms of total RNA, 1 µL oligo dT primer and certain volume of RNase-Free H_2_O were mixed into a total volume of 12 µL. The mixture was incubated at 70°C for 10 minutes and chilled on ice immediately. Four microliters 5×First Strand Buffer, 2 µL 0.1 M DTT, 1 µL 10 mM dNTP mix, and 1 µL SuperScript II were added to each tube and mixed. The tubes were incubated at 42°C for 50 minutes followed by 70°C for 15 minutes before chilling on ice. The cDNA was ready for PCR amplification. The PCR reactions were performed in a final volume of 20 µL in a Gene Amp- PCR system. The PCR reaction mix contained 1 µL of cDNA, 2 µL 10X PCR Buffer, 0.4 µL 10 mM dNTP mix, 1.2 µL 25 mM MgCl2, 1 µL 10 mM primer mix, 0.2 µL recombinant Taq polymerase, and 14.2 µL autoclaved ddH2O. The details of primer mixes used for each gene are as follows: β-actin: Forward ACA CCT CAA ACC ACT CCC AG; Reverse AAC TCC TAA GGG GAG GAT GG; p53: Forward GTGG ATCC TGAA GACT GGAT AACT GTC; Reverse AGTC GACA GGAT GCAG AGGC TG; MDM2: Forward GTCT CTGG ACTC GGAA GATT AC; Reverse AAAC AATG CTGC TGGA AGTC G.

For synthesis of β-actin gene as the internal control and Mdm2 gene, the PCR mixture was incubated at 94°C for 5 minutes followed by 30 cycles of amplification. Each cycle consisted of 45 seconds of denaturation at 94°C, 45 seconds of annealing at 55°C and 30 seconds of extension at 72°C. After all cycles were completed, a final extension step at 72°C for 10 minutes was performed. The PCR products were ready for gel electrophoresis. For synthesis of p53, the number of cycles was increased to 35, the annealing temperature was upped to 58°C, and the extension time was extended to 1.5 minutes.

### Immunodetection of Bax, Bcl-2 and Proliferation Cell Nuclear Antigen (PCNA) Protein Expression in Rat Livers

300 µg of each liver sample were homogenized in HEPES buffer(pH 7.9, 6% Triton X-100, 25% glycerol, 0.02 M HEPES, 0.2 mM EDTA, 61 mM NaCl, 1.2 mM MgCl_2_, 0.625 µM Dithiothreitol (DTT), 2.5 µM PMSF, Protease inhibitor cocktail (ROCHE)) at 1 tablet/50 mL using the glass homogenizer on ice. The homogenate was transferred to a microcentrifuge tube and centrifuged at 13,000 rpm for 20 minutes. The supernatant was collected and an equal volume of 2X sample loading dye was added to each sample. The samples were then stored at −20°C. The protein was used for immunodetection of the expression level of Bax, Bcl-2 and PCNA by Western blot analysis.

## Statistics

One-way ANOVA was performed to determine the statistical significance of differences among treated and untreated groups. Differences were considered statistically significant in all experiments at *p*<0.05 (*significantly different from untreated controls) or *p*<0.01 (**) level.

## Results and Discussion

**Figure 1:**
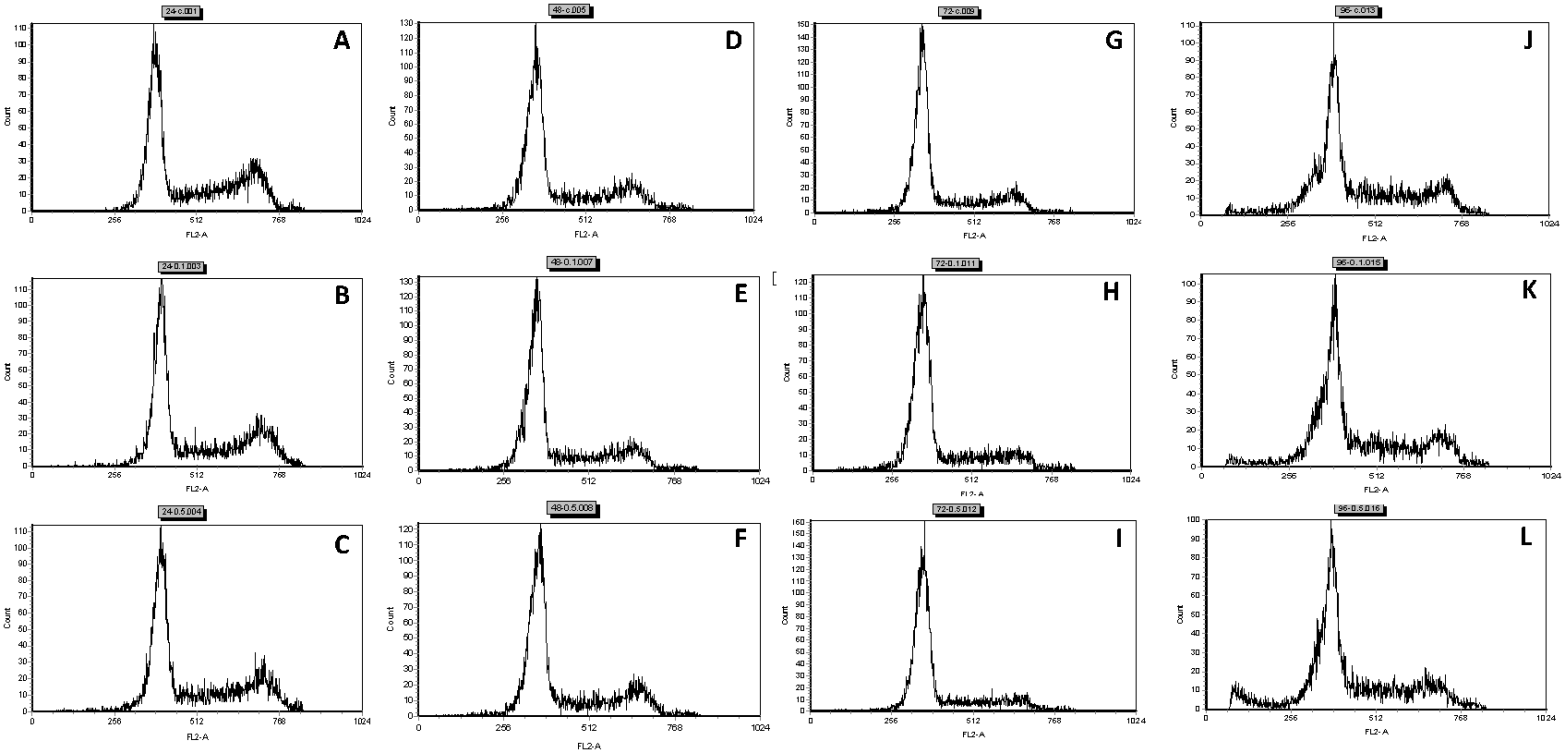
DNA Histograms demonstrating the attribution of HepG2 cells in cell cycle after sinigrin exposure for different periods of time. A: sinigrin, 0 mM/24 h; B: 0.1 mM/24 h; C: 0.5 mM/24 h; D: 0 mM/48 h; E: 0.1 mM/48 h; F: 0.5 mM/48 h; G: 0 mM/72 h; H 0.1 mM/72 h; I: 0.5 mM/72 h; J: 0 mM/96 h; K 0.1 mM/96 h; L: 0.5 mM/96 h.

**Figure 2:**
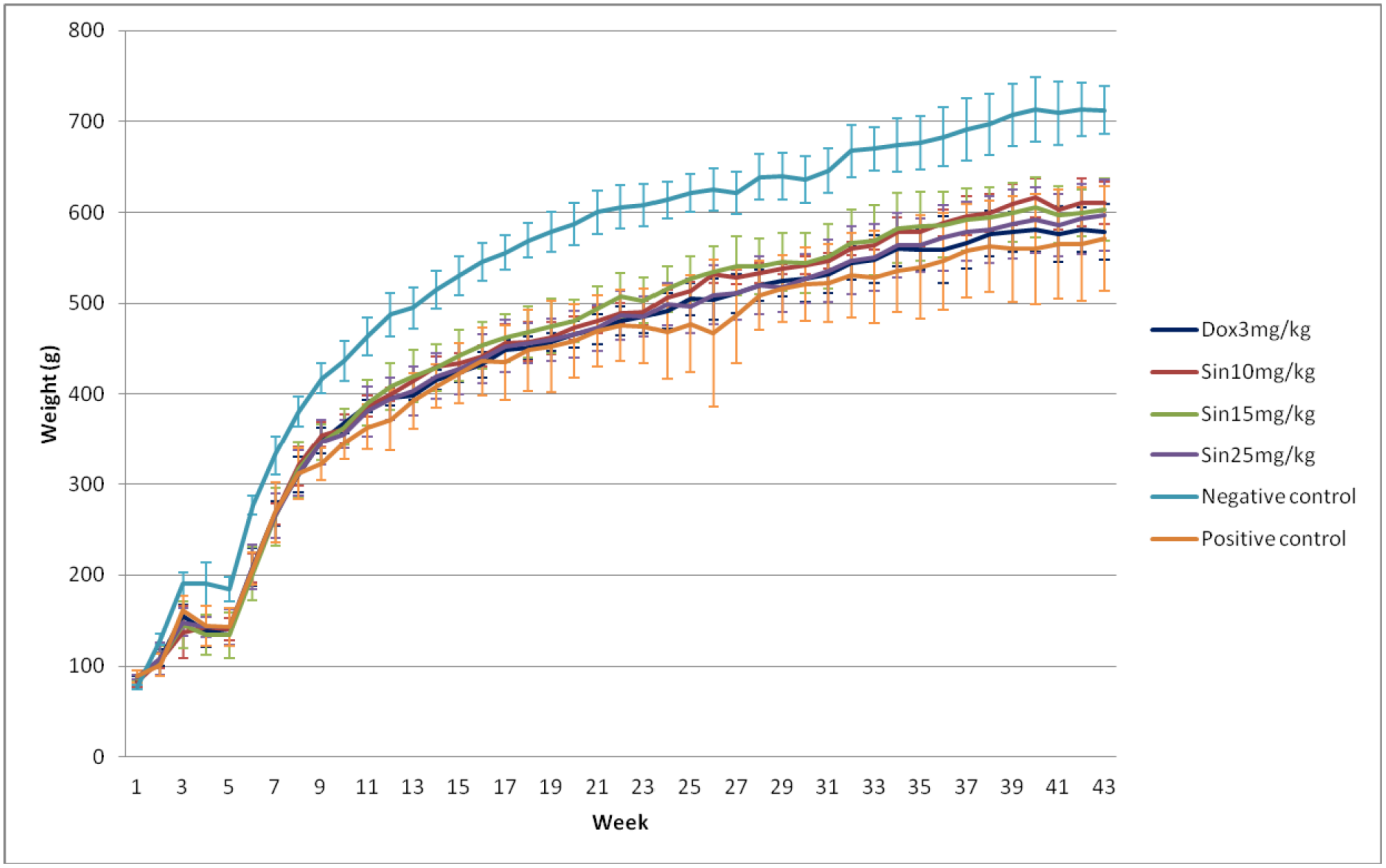
Change of body weight of rats in different control and treatment groups. Each value is expressed as mean ±SD (n = 10). Significance difference between control and treatment groups is indicated at *p*<0.05.

**Figure 3:**
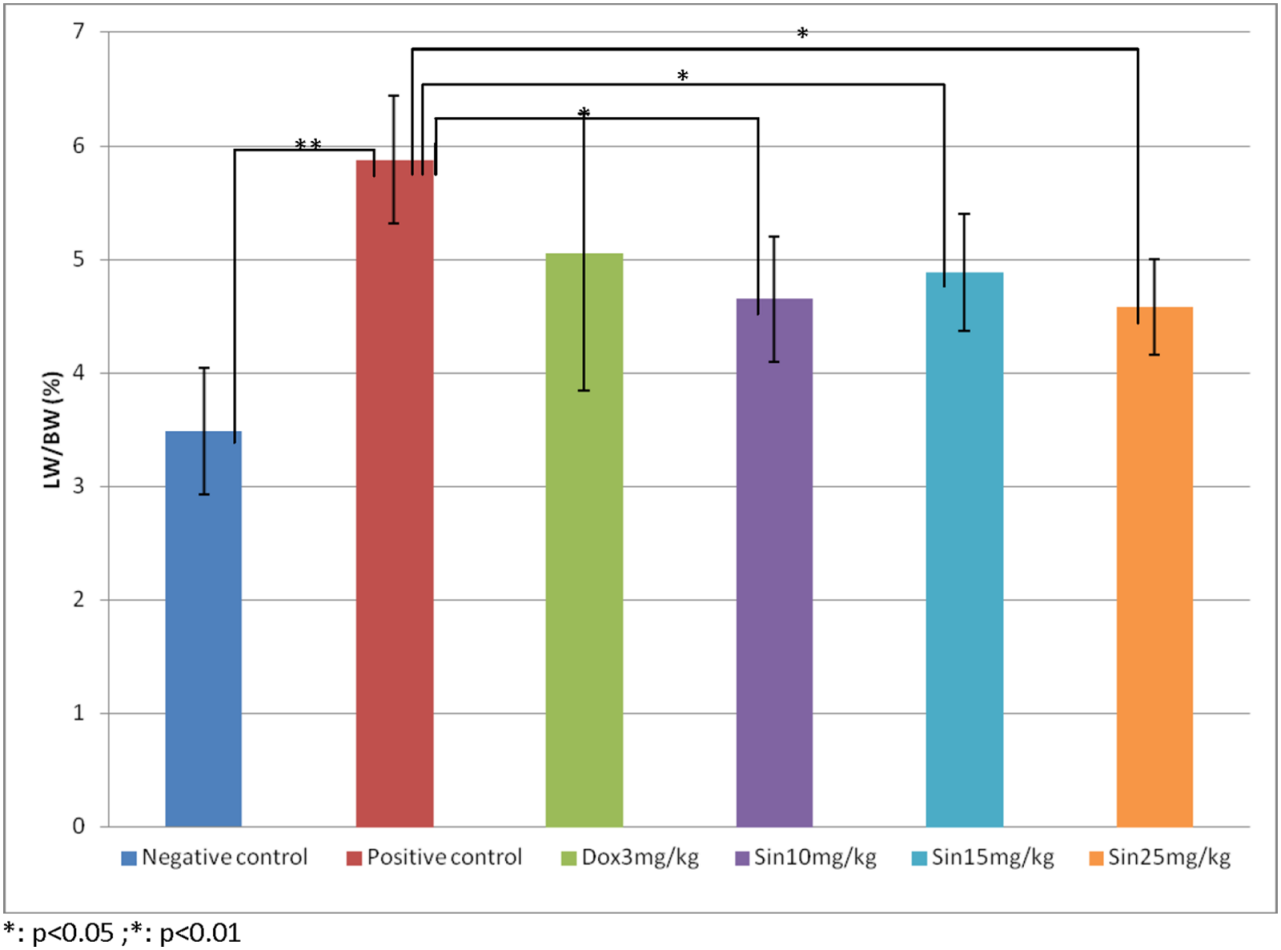
Effects of sinigrin treatment on liver weight/body weight ratio in different treatment groups. Data represent the mean ±SD (n = 10). Significance difference between treatment and control groups is indicated at *p*<0.05 (*) or *p*<0.01 (**) level.

**Figure 4:**
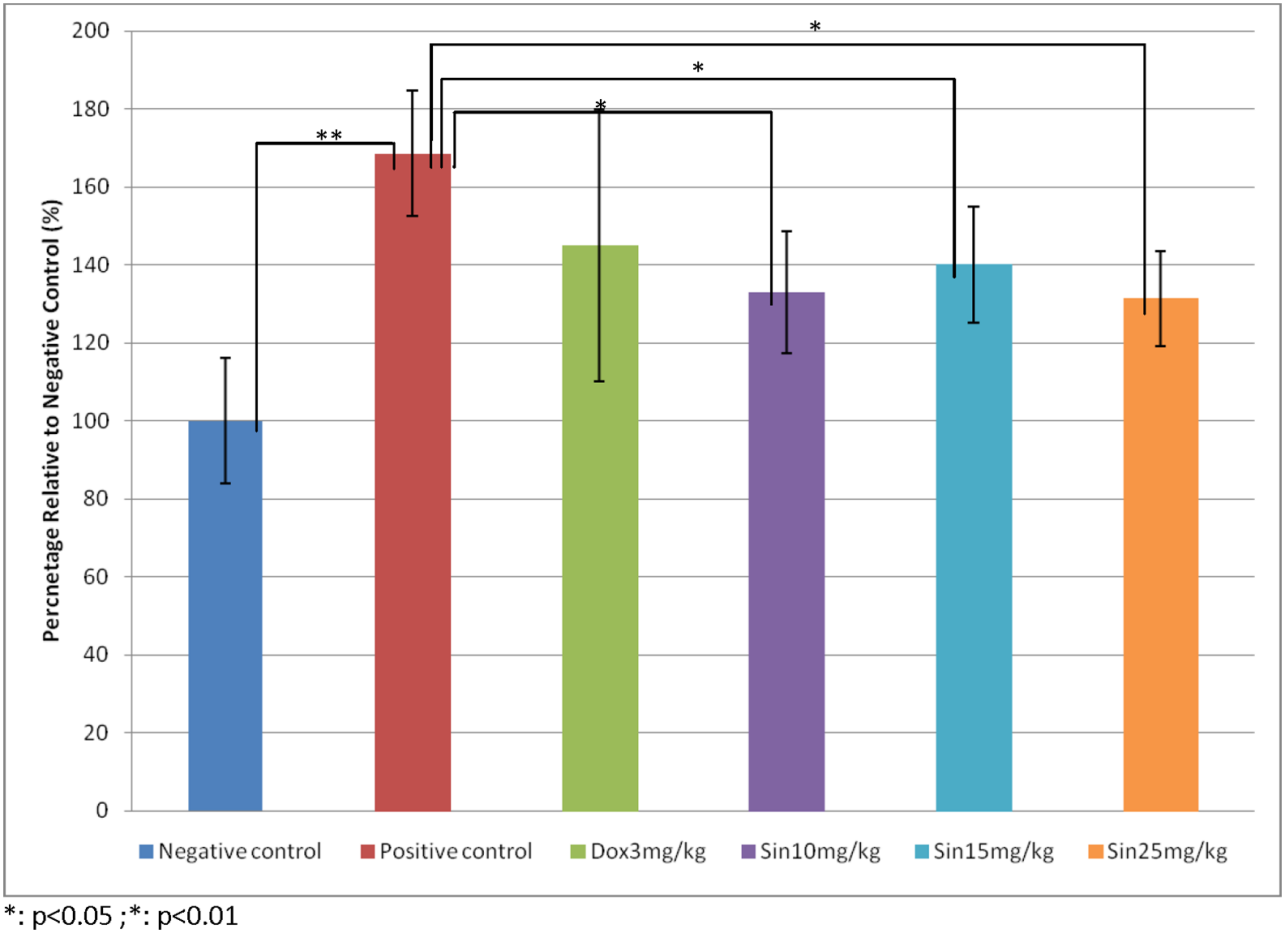
Change of liver weight index in treatment groups compared with the control group. Data represent the mean ±SD (n = 10). Significance difference between treatment and control groups is indicated at *p*<0.05 (*) or *p*<0.01 (**).

**Figure 5:**
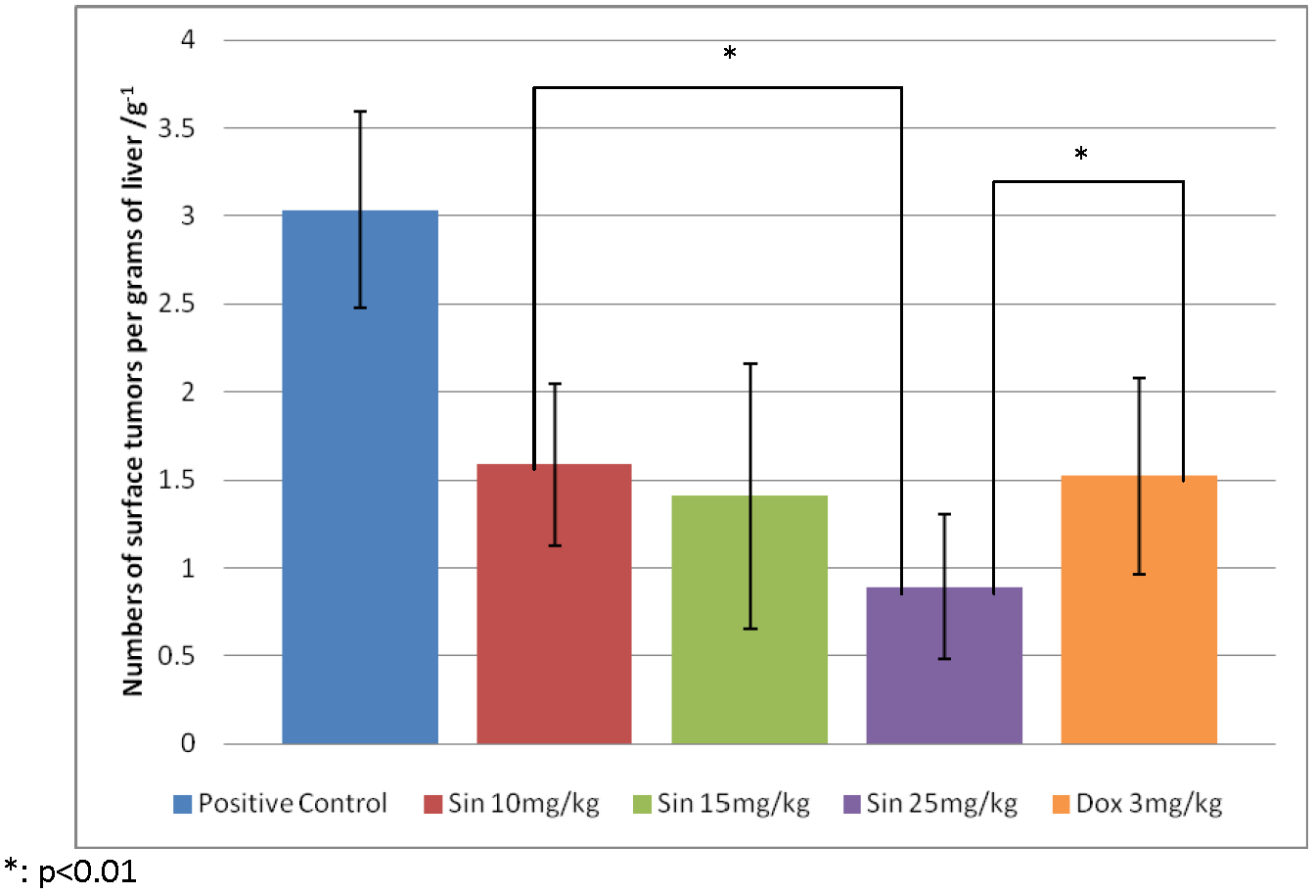
Effects of sinigrin treatment and doxorubicin treatment on the number of surface tumors in the rat. Data represent the mean ±SD (n = 10). Significant difference between treatment and control groups is indicated at *p*<0.01 (*).

**Figure 6:**
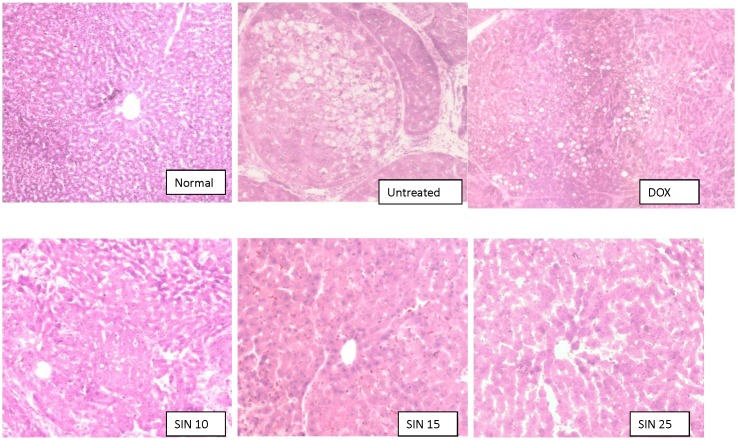
The hematoxulin-Eosin staining of rat liver sections from the control and treatment groups.

**Figure 7:**
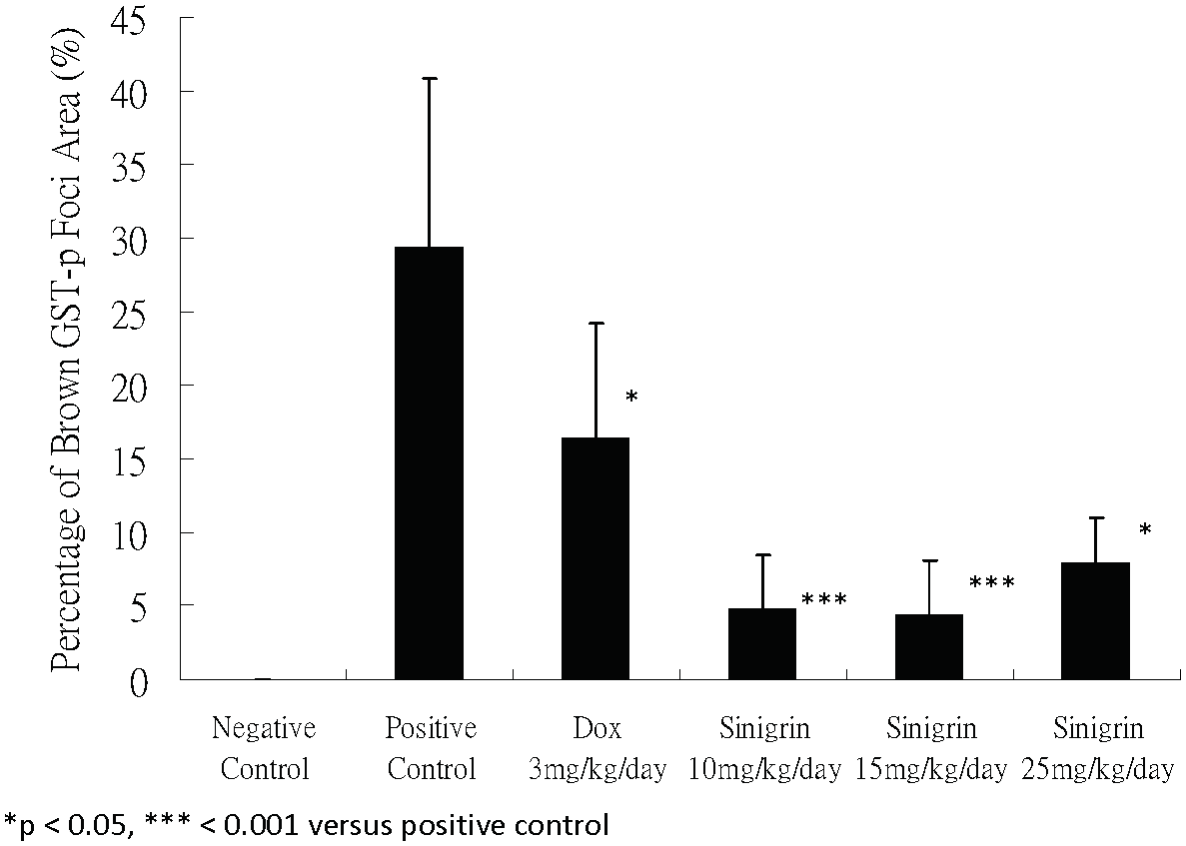
Effects of sinigrin treatment on the GST-p foci in the rat liver sections. Significance difference between treatment and control groups is indicated at *p*<0.05 (*) or *p*<0.001 (***).

**Figure 8:**
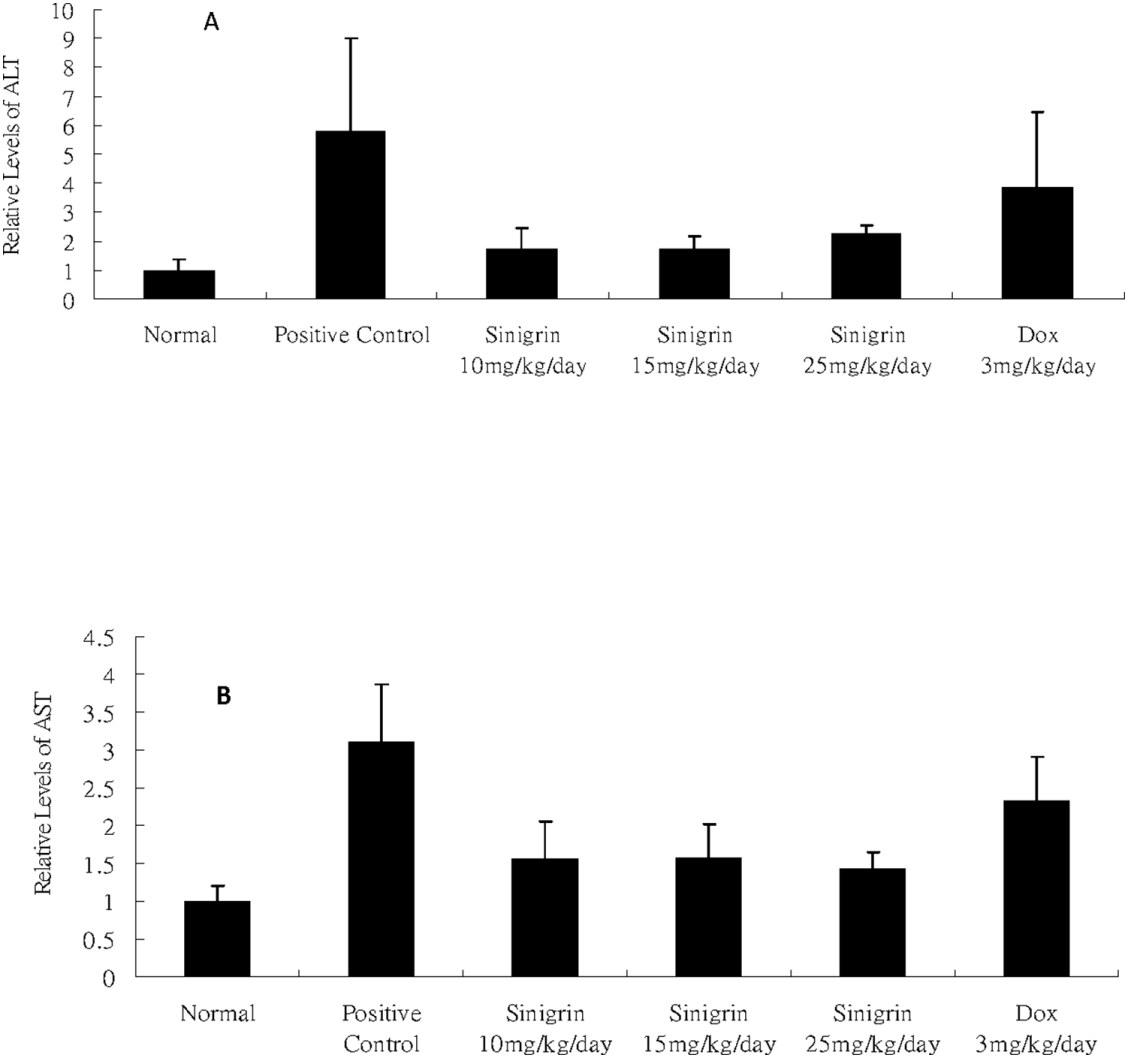
Effects of singrin treatment on the liver enzyme ALT & AST biomarkers among control and treatment groups. Data represent the mean ±SD (n = 3).

In this study, different dosages of sinigrin were administered to the rats following liver damage. An in vitro study on liver cancer cells revealed that sinigrin could induce apoptosis. Cell cycle analysis showed that sinigrin induced cell cycle arrest at G0/G1 phase (Figure 1). In vivo studies revealed that sinigrin triggers over-expression of p53 and down-regulation of Bcl-2 family members and caspases. The results suggest that sinigrin exhibited anti-tumor activity in the liver and are consistent with a previous study of the impact of sinigrin on cancer cell lines [12]. The inhibitory activity of sinigrin on carcinogen-induced liver damage was significantly attenuated. The gene expression of sinigrin-treated HepG2 cells revealed that sinigrin induced apoptosis via a p53-dependent pathway. This result is reminiscent of analogous in vitro studies of isothiocyanates [12]. In in vivo studies, rats after treatment with sinigrin showed an obvious change of body weight in different control and treatment groups (Figure 2). The body weight of the positive control group was significantly reduced. The results suggest the liver function of the positive control group (untreated rats having liver cancers) was attenuated compared with the treatment group. Effects of sinigrin treatment on the liver weight of different groups of rats are shown in Figure 3. The results showed that the liver weight of the positive control group was significantly increased whereas that of the treated group was reduced after treatment with sinigrin. These studies reflect the health benefits of sinigrin towards carcinogens-induced liver injury. The change in liver weight index (The ratio of liver weight/body weight was compared with that of a normal rat) in different treatment groups and the control groups are shown in Figure 4. These results indicate that the liver weight index of treatment groups of rats was reduced compared with that of the negative control group and the positive control group. The liver/body weight ratio showed the steatotic or cirrhotic levels in the rat liver. In order to compare the efficacy of sinigrin with a common cancer drug, doxorubicin was used to treat the carcinogens-induced hepatocarcinogenesis in the rat. The results showed that after doxorubicin treatment the number of surface tumors in the rat liver was not affected whereas the group of rats that was treated with sinigrin displayed considerably reduced numbers of surface tumors (Figure 5). The liver weight index of doxorubicin-treated group was similar to that of the positive control group. The histological changes of liver section from rats were examined. The results indicated that there were obvious histological changes between treated and the untreated groups of rats (Figure 6). The liver sections from doxorubicin-treated rats displayed similar structures to those of the positive control groups of rats. These results on histological change suggest that doxorubicin was not effective at altering carcinogen rats at this stage of tumor development. The liver sections of different control and treatment groups of rats were also analyzed with GST-p antibodies (Figure 7). The results of GST-p antibody labeling of the assorted liver sections provide supportive experimental evidence that rat liver functions could be restored after treatment with sinigrin relative to the negative control and the positive groups of rats. The GST-p foci showed clearly that doxorubicin treatment was ineffective managing or attenuating carcinogen-induced hepatocarcinogenesis. The levels of the rat liver enzymes ALT and AST were, importantly, significantly decreased (Figure 8). These data suggest that liver functions can be gradually restored after treatment with sinigrin.

**Figure 9:**
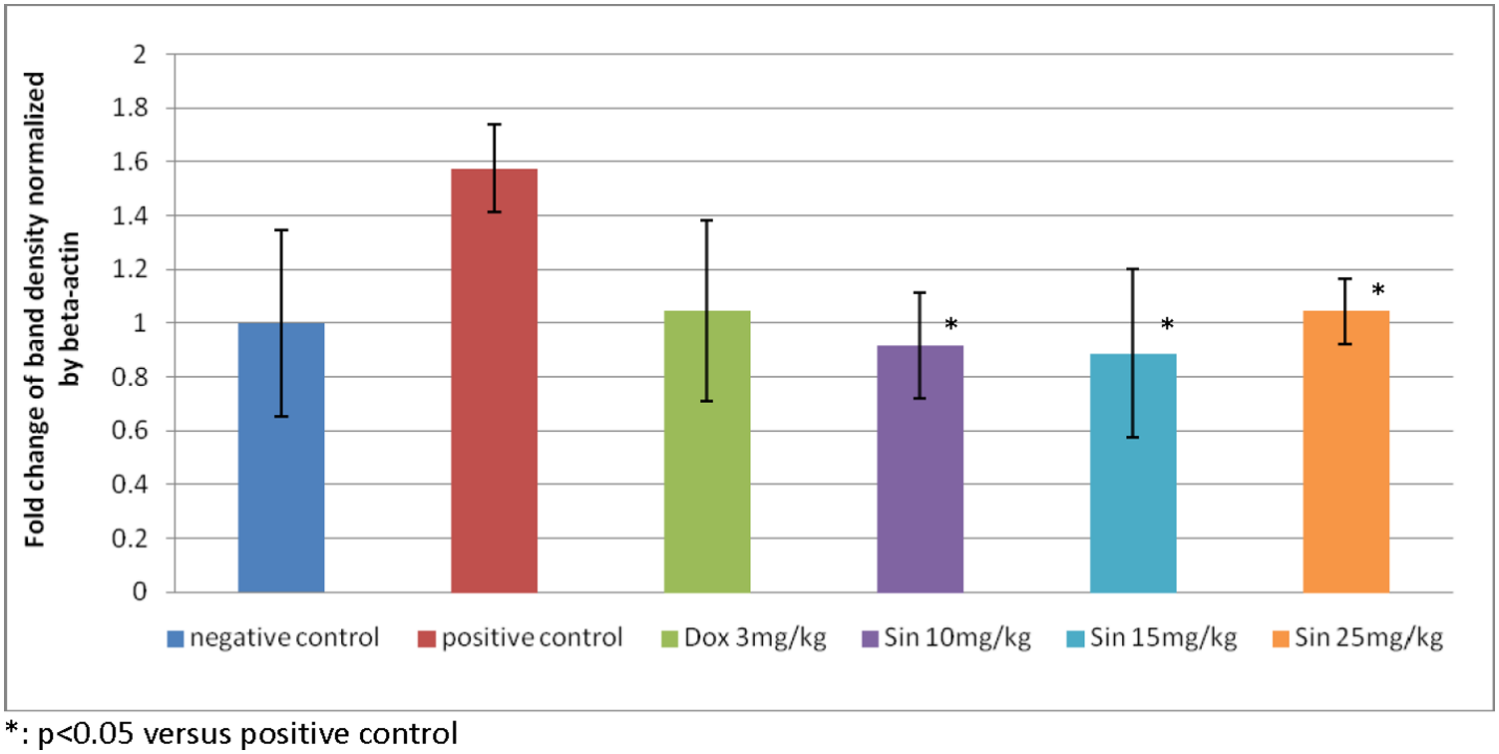
Fold change of p53 mRNA expression in different treatment groups. The immunoblots shown here are representative of three independent experiments with similar results.

**Figure 10:**
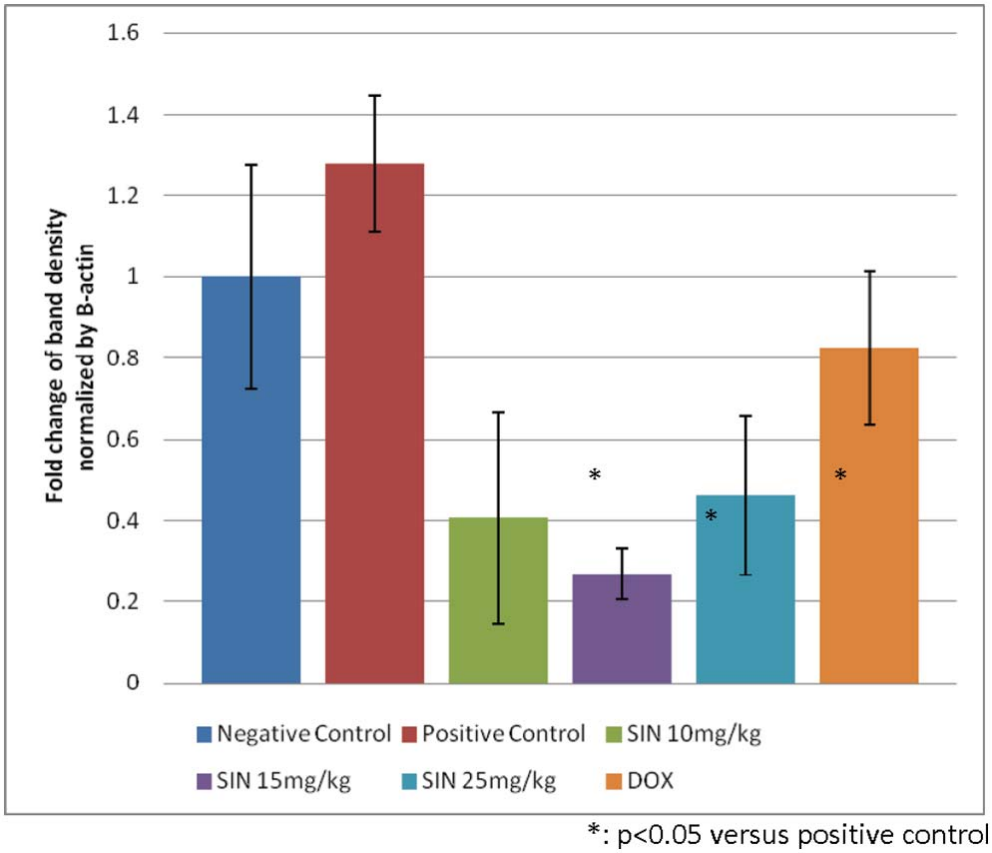
Change of MDM2 mRNA expression in treatment group compared with the control. The immunoblots shown here are representative of three independent experiments with similar results.

**Figure 11:**
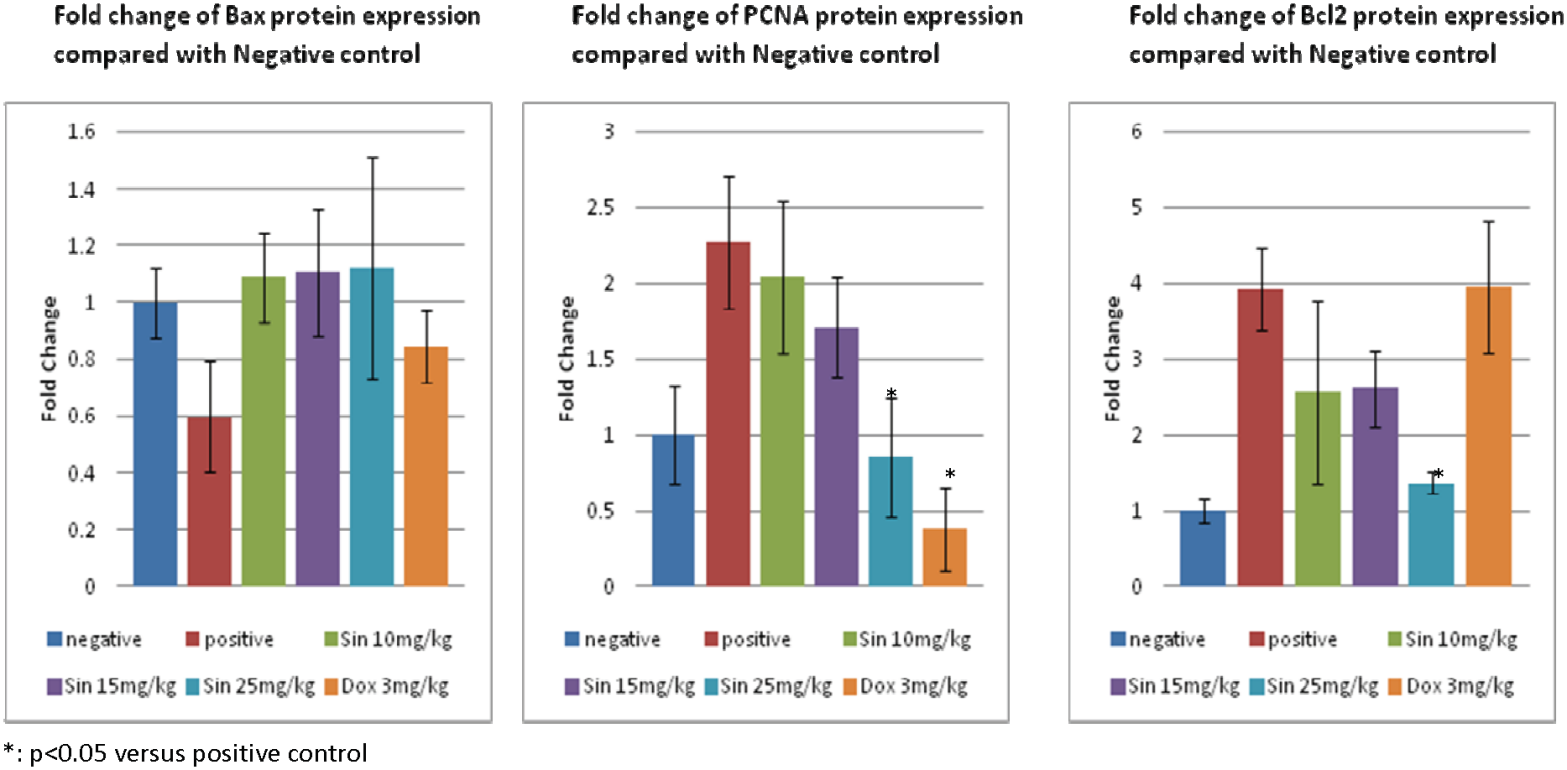
Change of Bax, Bcl-2, PCNA protein expression between treatment group and the control group. The immunoblots shown here are representative of three independent experiments with similar results.

At the molecular level, an attempt was made to understand possible changes in gene expression associated with sinigrin treatment. The results, as indicated in Figure 9, revealed profound changes in the levels of p53 mRNA in rats following treatment with sinigrin. This change reflects the likelihood that sinigrin induces apoptosis via a p53-dependent pathway. Notably, levels of MDM2 mRNA in the sinigrin treatment group also were considerably higher than in the negative control group (Figure 10). This change in MDM2 expression was accompanied by alterations in the gene expression of Bax, Bcl-2 and PCNA (Figure 11).

These findings suggest that sinigrin exerted anti-proliferative activity carcinogen-induced liver damage in the rat, and caused cell cycle arrest and amelioration of liver functions in the rat. The anti-proliferative activity of sinigrin was increased with increasing dosages. The present findings demonstrate that sinigrin is an excellent glucosinolate with medicinal properties, and add to the literature on bioactive phytochemicals in cruciferous vegetables, especially glucosinolate compounds that can inhibit the growth of liver cancer cells [1]–[4]; such information pertaining to the anti-cancer activity of the glucosinolates endogenous to Brassica is limited. Sinigrin was shown to possess anti-cancer activities in the present study. The cell cycle analysis indicated that sinigrin caused cell cycle arrest in G0/G1 phase (Figure 1). Sinigrin triggered the release of cytochrome c through the down-regulation of Bcl-2 and up-regulation of Bax. The present findings reveal that Bcl-2 protein expression was significantly lower in the sinigrin treated group of rats than in the positive control group. Bax was over-expressed in the negative control and the sinigrin-treated groups relative to the positive control group. These results suggest that the over-expression of mutant p53 was increased in the rat after carcinogen exposure and subsequent carcinogenesis. The major regulator of p53 turnover, Mdm2 was found to be over-expressed at the transcriptional level in the untreated positive controls. The over-expression of Mdm2 is associated with the wild-type p53 degradation. The significant increase in the expression of p53 mRNA in the positive control rats lead to an increase in total p53 protein during carcinogenesis, whereas the mRNA expression was decreased in the sinigrin-treated group of rats. These findings suggest that sinigrin down-regulates the expression of Mdm2 thus leading to an increase in p53 expression. The over-expression p53 presumably enhances expressions of Bax, Bcl-2 and p21 leading to cell cycle arrest and apoptosis. Notably, the positive control group of rats was found to show a considerable increase in PCNA expression whereas the sinigrin-treated rats showed a decrease in PCNA expression. This suggests that liver cell proliferation was attenuated by subjection to sinigrin. These findings suggest that liver damage related to carcinogen triggered carcinogenesis was remarkably reduced in rats following exposure to sinigrin. Indeed, the histological changes observed between different treatment groups and the negative control groups reveal that sinigrin appears to attenuate carcinogen-triggered liver injury in the rat.

Histological changes in liver sections from the treated groups of rats suggest that sinigrin suppresses the formation of preneoplastic foci due to carcinogen exposure. Gross examination of the liver from the positive control rats showed the presence of lipid droplets, nodules and tumors across the surface of the rat liver. Histological examination of rat liver sections revealed that hepatocytes of sinigrin -treated rats appeared to display the cuboidal shape that is characteristic of normal hepatocytes. These findings demonstrate that sinigrin exhibits anti-cancer properties in the rat on a dosage dependent manner. The health benefits of sinigrin was confirmed when a higher dose of sinigrin was used. The results suggest that the anti-cancer activity of sinigrin is attributed to the radical scavenging capacity of sinigrin which is believed to play an important role in ameliorating liver damage [13]–[14].

The ratio of liver weight and body weight serves as a representative marker of the anti-oxidative capacity of the liver. A decrease in ALT and AST suggests risk reduction for oxidative damage. Alterations in the enzyme levels for ALT and AST are common features of oxidative stress and pathological conditions associated with liver damage [15]. Reductions of ALT and/or AST levels are important for instilling protection from carcinogen-induced liver damages. In the present study, we showed that treatment with sinigrin remarkably decreased the GST-p foci which were greater than those observed for the untreated group and the doxorubicin-treated group. This suggests that sinigrin possesses anti-cytotoxic activities in carcinogen-induced liver injury.

Previous studies have revealed that cells differ in their response to carcinogens depending on their levels of p53 activity [16]. Activation of p53 in liver cells resulted in up-regulation of p21 and to increased expression of Bax and, proapototic Bcl-2 activities leading to mitochondrial pore opening. Both p53 and p21 protein levels were also increased. The activation of p53 plays a crucial role in sinigrin-induced down-regulation of Bax, Mdm2 and Bcl-2 family members highlighting that a functional p53 is required for the execution of apoptosis in cancer cells.

It is known that p53 regulates the cell cycle and apoptosis by activating expression of target proteins including Mdm2, p21, PCNA, Bcl-2 and Bax. Mdm2 is associated with the regulation of p53 turnover [17]. The over-expression of Mdm2 protein likely enhances degradation of more wild type p53 protein. Importantly, Mdm2 was found to be over-expressed in the positive control group whereas the expression levels of Mdm2 were down-regulated in sinigrin-treated cells relative to the positive control. These results suggest that sinigrin is capable of down regulating the critical regulator Mdm2. In a related fashion, increases in p21 are known to be related to the inactivation of PCNA. Importantly, PCNA levels examined in these studies were found to be lower in the sinigrin-treated group than in the positive control groups, suggesting that cell division also, is a target for sinigrin-dependent attenuation.

## Conclusion

The present study demonstrates that sinigrin can inhibit cancer growth through a p53-dependent pathway. Sinigrin can improve the liver functions and reduce the tumor burden. The changes in gene expression after sinigrin treatment provide experimental evidences that sinigrin is capable of mediating cell cycle arrest through apoptotic events. Sinigrin does not cause liver toxicity in rats. The anti-proliferative activities of sinigrin on carcinogens-induced liver carcinogenesis were demonstrated in the present study.

## References

[1] Rungapamestry V, Rabot S, Fuller Z, Ratcliffe B, Duncan AJ (2008) Influence of cooking duration of cabbage and presence of colonic microbiota on the excretion of N-acetylcysteine conjugates of allyl isothiocyanate and bioactivity of phase 2 enzymes in F344 rats. British Journal of Nutrition 99: 773–781. 17967216 10.1017/S0007114507841134

[2] Krul C, Humblot C, Philippe C, Vermeulen M, van Nuenen M, et al (2002) Metabolism of sinigrin (2-propenyl glucosinolate) by the human colonic microflora in a dynamic in vitro large-intestinal model. Carcinogensis 23: 1009–1016. 10.1093/carcin/23.6.1009 12082023

[3] Smith TK, Lund EK, Clarke RG, Bennett RN, Johnson IT (2005) Effects of Brussels sprout juice on the cell cycle and adhesion of human colorectal carcinoma cells (HT29) in vitro. Journal of Agricultural and Food chemistry 53: 3895–3901. 15884814 10.1021/jf048025v

[4] Munday R, Munday CM (2002) Selective induction of phase II enzymes in the urinary bladder of rats by allyl isothiocynate, a compound derived from Brassica vegetables. Nutrition and Cancer 44: 52–59. 12672641 10.1207/S15327914NC441_7

[5] Vines G (1996) My best friend’s a Brussels sprout. New Scientist 2061: 46–49.

[6] TawFig N, Heaney RK, Plumb JA, Fennich GR, Musk SRR, et al (1995) Dietary glucosinolates as blocking agents against carcinogenesis: glucosinolate breakdown products assessed by induction of quinone reductase activity in murine hepa1c1c7 cells. Carcinogenesis 16: 1191–1194. 7767984 10.1093/carcin/16.5.1191

[7] Takuji T, Yoshio M, Yukio M, Akira H, Takatoshi O, et al (1990) Inhibitory effect of sinigrin and indole-3-carbinol on diethylnitrosamine-induced hepatocarcinogenesis in male ACI/N rats. Carcinogenesis 11: 1403–1406. 2387027 10.1093/carcin/11.8.1403

[8] Okulicz M (2010) Multidirectional time-dependent effect of sinigrin and allyl isothiocyanate on metabolic parameters in rats. Plant Foods for Human Nutrition (Dordrecht) 65: 217–224. 10.1007/s11130-010-0183-3 PMC2944953 20809411

[9] Mays JR, Roska RLW, Sarfaraz S, Mukhtar H, Rajski SR (2008) Identification, synthesis, and enzymology of non-natural glucosinolate chemopreventive candidates. ChemBioChem 9: 729–747. 18327862 10.1002/cbic.200700586

[10] Munday R, Munday CM (2002) Selective induction of phase II enzymes in the urinary bladder of rats by allyl isothiocyanate, a compound derived from Brassica vegetables. Nutrition and Cancer 44: 52–59. 12672641 10.1207/S15327914NC441_7

[11] Rocha NS1, Barbisan LF, de Oliveira ML, de Camargo JL (2002) Effects of fasting and intermittent fasting on rat hepatocarcinogeneis induced by diethylnitrosamine. Teratog Carcinog Mutagen. 22(2): 129–138. 10.1002/tcm.10005 11835290

[12] Pitot HC (1990) Altered hepatic foci: Their role in murine hepatocarcinogenesis. Annu Rev Pharmacol Toxicol 30: 465–500. 2188576 10.1146/annurev.pa.30.040190.002341

[13] McGhee JO’D, Patrick RS (1969) The synthesis of sulphated mucopolysaccharide in mouse liver following carbon tetrachloride injury. I. Autoradiographic studies. Br J Exp Pathol 50: 521–526. 4243672 PMC2072166

[14] Slamenova D, Kozics K, Hunakova L, Melusova M, Navarova J, et al (2013) Comparison of biological processes induced in HepG2 cells by tert-butyl hydroperoxide (t-BHP) and hydroperoxide (H_2_O_2_): The influence of carvacrol. Mutat Res 757: 15–22. 23867853 10.1016/j.mrgentox.2013.03.014

[15] Navarro-Yepes J, Burns M, Anandhan A, Khalimonchuk O, Del Razo LM, et al.. (2014) Oxidative stress, redox signaling and autophagy: Cell death vs Survival. Antioxid Redox Signal (In press). 10.1089/ars.2014.5837 PMC4048575 24483238

[16] Ostrakhovitch EA, Cherian MG (2005) Role of p53 and reactive oxygen species in apoptotic response to copper and zinc is epithelial breast cancer cells. Apoptosis 10: 111–121. 15711927 10.1007/s10495-005-6066-7

[17] Power C, Sinha S, Webber C, Manson MM, Neal GE (1987) Transformation related to expression of glutathione S-transferase P in rat liver cells. Carcinogenesis 8: 797. 3111738 10.1093/carcin/8.6.797

